# Task Offloading Strategy Based on Mobile Edge Computing in UAV Network

**DOI:** 10.3390/e24050736

**Published:** 2022-05-22

**Authors:** Wei Qi, Hao Sun, Lichen Yu, Shuo Xiao, Haifeng Jiang

**Affiliations:** 1 Department of Information Technology, Jiangsu Union Technical Institute, Xuzhou 221000, China; ts20170065p31@cumt.edu.cn; 2 School of Computer Sciences and Technology, China University of Mining and Technology, Xuzhou 221000, China; ts20170094p31@cumt.edu.cn (H.S.); sxiao@cumt.edu.cn (S.X.); jhfeng@cumt.edu.cn (H.J.); 3 School of Sciences, Xi’an Jiaotong-Liverpool University, Suzhou 215123, China

**Keywords:** unmanned aerial vehicle, mobile edge computing, stackelberg game

## Abstract

When an unmanned aerial vehicle (UAV) performs tasks such as power patrol inspection, water quality detection, field scientific observation, etc., due to the limitations of the computing capacity and battery power, it cannot complete the tasks efficiently. Therefore, an effective method is to deploy edge servers near the UAV. The UAV can offload some of the computationally intensive and real-time tasks to edge servers. In this paper, a mobile edge computing offloading strategy based on reinforcement learning is proposed. Firstly, the Stackelberg game model is introduced to model the UAV and edge nodes in the network, and the utility function is used to calculate the maximization of offloading revenue. Secondly, as the problem is a mixed-integer non-linear programming (MINLP) problem, we introduce the multi-agent deep deterministic policy gradient (MADDPG) to solve it. Finally, the effects of the number of UAVs and the summation of computing resources on the total revenue of the UAVs were simulated through simulation experiments. The experimental results show that compared with other algorithms, the algorithm proposed in this paper can more effectively improve the total benefit of UAVs.

## 1. Introduction

Power patrol inspection includes calculation-intensive tasks such as fault identification and foreign-object detection. However, such tasks require UAVs to carry out complex calculations in a limited time [1]. Due to the limited battery energy and storage space, the efficiency of UAVs is low. To solve this problem, some researchers have proposed transmitting the computing tasks or data from mobile devices to the remote cloud for execution [2]. However, traditional cloud computing usually connects the remote cloud center with a large transmission delay and an unstable wireless connection, which cannot meet the real-time needs of users [3]. Different from traditional cloud computing technology, mobile edge computing is a new technology. This offloads the computing tasks from mobile devices to the network edge cloud for real-time data transmission and computing, thus expanding the capabilities of mobile devices [4,5]. In this paper, the MEC task offloading part is added to the UAV patrol system, and the distributed computing method is adopted to sink the computing task to the edge of the network, so as to reduce the demand for equipment to offload data to the cloud server and effectively reduce network congestion and delay [6,7].

In research on UAV-aided wireless communication systems with edge computing, the optimization problems and constraints change with the change in the UAV application scenario. For example, Li et al. [8] considered a scenario where a UAV with edge computing functions assists ground IOT equipment in data acquisition and calculation and jointly optimizes UAV trajectory, communication bandwidth allocation, and calculation offloading strategy based on the goal of minimizing the total energy consumption of IOT equipment. In [9], Liu et al. studied a scenario in which multiple UAVs assist ground IOT equipment in computing and offloading. The authors also took UAV energy consumption into account to minimize the total energy consumption of UAVs and ground equipment. In research on UAV-assisted ground mobile users in computing offloading, Jeong [10], Hu [11], and Xiong [12] carried out UAV trajectory planning and computing offloading strategy allocation from the perspective of system energy consumption optimization. Different from the above research studies, Hu Q et al. assumed that the ground user can calculate some tasks locally and then offload the remaining tasks to the UAV in the scenario where the ground user uses the UAV to perform remote computing, and the optimization goal is to minimize the maximum transmission delay of all users [13]. In the study [14], Wang et al. proposed a heuristic-calculation offloading algorithm.

Zhou [15] studied a new system whereby a UAV assists wireless power transmission with edge computing, in which UAVs can not only transmit energy signals to ground mobile users but can also provide users with computing offloading. The author optimized a UAV trajectory and computing offloading strategy based on the goal of maximizing the total energy of users considering fairness; in [16], the author further studied the system from the perspective of minimizing UAV energy consumption. In addition, some intelligent optimization algorithms have also been introduced into research on wireless communication systems aided by UAVs. For example, Wan et al. [17] proposed a new online computing offloading algorithm based on Lyapunov optimization in research on multi-UAV-assisted computing offloading of ground IOT equipment. Wang et al. [18] proposed a user scheduling and computing offloading algorithm based on reinforcement learning in research on multi-UAVs as a relay to provide computing offloading for ground mobile users.

Research on the networked UAV communication system with edge computing usually focuses on the differences among the offloading modes of UAVs. Cao et al. [19] creatively proposed a new scheme whereby networked UAVs can enhance their own computing performance by using edge computing technology by studying the application scenario where multiple ground base stations assist networked UAVs to perform computing offloading. Aiming at minimizing the task completion time of UAVs, the UAV trajectory and offloading strategy are jointly optimized. Based on the consideration of transmission delay and energy constraints, Ateya et al. [20] studied a scenario where networked UAVs can choose to offload computing-intensive tasks to ground base stations or other UAVs nearby. In [21], Chen et al. proposed an intelligent UAV computing offloading algorithm based on a deep Monte Carlo tree search in the study of ground base stations assisting multiple UAVs to perform computing task offloading.

As the ground base station has the advantages of sufficient computing resources and convenient energy supply, authors usually focus on the optimization of UAV energy consumption in research on the energy consumption of networked UAV communication systems with edge computing. For example, Fan et al. [22] considered a scenario where a simple UAV offloads to a ground base station. Assuming that the UAV itself could complete some local calculations, the authors minimized the flight energy consumption of the UAV by optimizing the UAV trajectory. Hua et al. [23] studied a scenario in which a ground base station assists multi-vehicle UAVs to perform computing offloading. With various UAV access schemes, such as time-division multiple access, orthogonal-frequency-division multiple access, and nonorthogonal-frequency-division multiple access, the authors jointly optimized the UAV trajectory, transmit power, and computing offloading strategy with the goal of minimizing the total energy consumption of UAVs. It is worth noting that, in practical applications, when the networked UAV offloads some confidential computing tasks to the ground base station, some important information is likely to be intercepted by the ground eavesdropper. Therefore, research on secure-communication-oriented networked UAV communication systems with edge computing is also very important. Bai et al. [24] considered a scenario where a fixed UAV offloads some computing tasks to the ground base station in the presence of ground eavesdroppers. The authors optimized the computing offloading strategy with the goal of minimizing the total energy consumption of UAVs.

In [25], Avgeris et al. presented a three-level cyber-physical social system (CPSS) for early fire detection to assist public authorities in promptly identifying and acting on emergency situations; they designed a dynamic resource scaling mechanism for the edge computing infrastructure, which can address the demanding Quality of Service (QoS) requirements of this IoT-enabled time and mission-critical application.

To sum up, in existing research on UAV-aided wireless communication systems with edge computing, authors have mainly focused on the optimization of system energy consumption and constraints on transmission delay caused by computing offloading. For the networked UAV communication system with edge computing, the computing power of the ground base station is generally much greater than that of the UAV or other equipment, and the transmission delay problem can usually be ignored. Therefore, existing research studies have mainly focused on optimizing the total task time or total energy consumption of the UAV.

In this article, to balance the delay and consumption of UAVs and optimize the process of computing offloading in UAV networks, we utilize the Stackelberg game model to model the UAV network. Then, based on the game model, to deal with the complex UAV-MEC task offloading problem, the Markov Decision Process (MDP) and the MADDPG algorithm are introduced to solve the resource allocation interaction model of the UAV and MEC. Extensive experiments are performed to evaluate and compare the performance of the MADDPG and related algorithms, and the results verify the effectiveness of the proposed algorithm in reducing the delay and energy consumption of UAVs and maximizing the utility of UAV networks.

## 2. System Model

### 2.1. System Architecture

**Figure 1:**
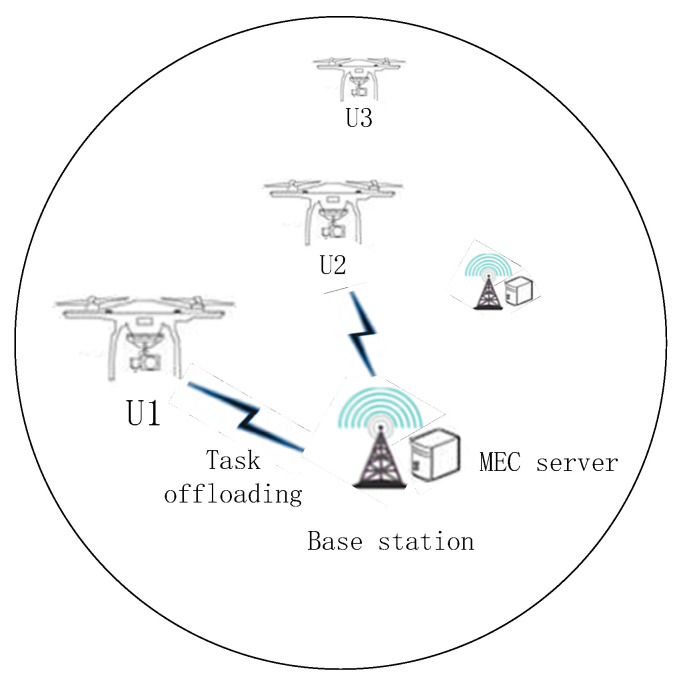
Task offloading system model in UAV network.

As shown in Figure 1, we consider a scenario where the edge node is composed of a base station (BS) responsible for communication and a MEC server that can provide computing services serves multiple patrol UAVs; the communication between UAVs and edge nodes adopts an orthogonal-frequency-division multiple access system, whereby each channel is orthogonal to the others. Each UAV can only be assigned to one channel, so interference can be avoided.

It is assumed that there are N UAVs in the system, the set of which is $U=\left\{u_{1},u_{2},\cdot \cdot \cdot ,u_{n}\right\}$, and the UAVs are randomly distributed around the edge node along the line. There are M edge nodes in the system, and the set of edge nodes is $V=\left\{v_{1},v_{2},\cdot \cdot \cdot ,v_{m}\right\}$. Each UAV generates tasks randomly and can purchase computing resources from edge nodes. The computing resource that edge nodes v can provide is $f_{v}$. We consider a quasi-static scenario, that is, the UAV moves in different periods, and its position remains unchanged for a period of time [26].

### 2.2. Two-Stage Stackelberg Game Model

**Figure 2:**
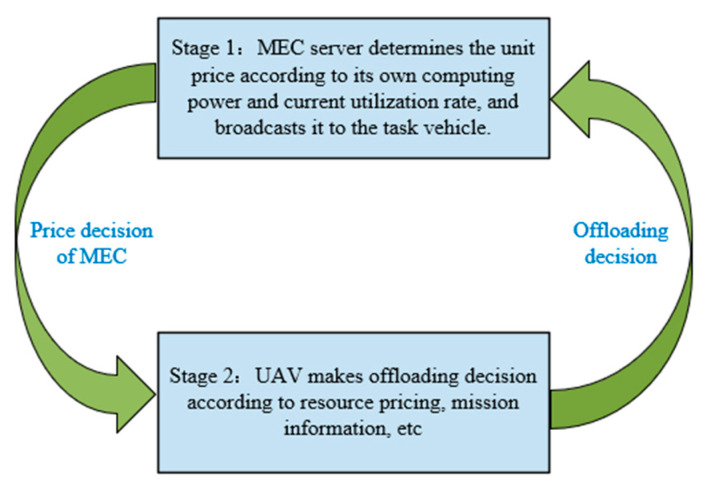
Two-stage Stackelberg game model.

We construct a two-stage Stackelberg game model, as shown in Figure 2. In the first stage of the game, the MEC server determines the unit price of its computing resources according to its own computing resources and current utilization rate and broadcasts it to the UAV. In the second stage, the UAV decides which edge nodes to offload to and how much computing resources to purchase, as well as what proportion to offload according to its own price-sensitive factor; delay-sensitive factor; task priority-, task success-, or failure-sensitive factor; task information.

### 2.3. Communication Model

The transmission rate [27] $r_{u,v}$ from UAV u to MEC server v can be expressed as follows:

(1) $$r_{u,v}=\omega \log_{2}(1+a_{u,v}\cdot P_{u}^{T}),a_{u,v}=\frac{\sigma_{c}|h_{u}|}{\Gamma d^{\lambda }N_{0}}$$

where ω represents the bandwidth, due to the total bandwidth B being divided into H channels, so ω=B/H. $P_{u}^{T}$ indicates the transmission power of UAV u. The Rayleigh channel model with shadow fading coefficient $\sigma_{c}$ and noise $N_{0}$ power is adopted. The parameters Γ in the channel model represent the signal-to-noise ratio to ensure the minimum bit error rate. It is assumed that the channel coefficient $h_{u}$ is perfectly estimated, and the channel fading is constant throughout the transmission cycle. λ is the path loss index. d represents the distance between UAV u and edge node v.

### 2.4. Task Model

The task in the system is recorded as T. It is assumed that the task can be divided and offloaded to different edge nodes for parallel computing. The data volume of the task T is D bits. Generally, the index to measure the amount of computation of a task is the number of CPU cycles, which can be calculated by c=αD, where α represents the number of CPU cycles required when calculating one-bit data, which is determined by the task type. For complex tasks, α is usually large. Usually, the return result of the task is much smaller than the input data of the task. Task T can be represented by a triple $\langle D,\alpha ,t^{\max}\rangle$.

### 2.5. Computation Model

This section proposes a computational model to represent the execution time of tasks on local and MEC servers. Each UAV can be represented as a quad $\langle x_{0},y_{0},t,f_{local}\rangle$; $x_{0}$ and $y_{0}$ represent the position of the UAV in the system; t is the time of the UAV in the system; $f_{local}$ is the computing power of the UAV.

Local execution time can be expressed as follows:

(2) $$t^{all\_local}=\frac{\alpha D}{f_{local}}$$

The process of offloading tasks to edge nodes can be divided into the following three stages: offloading, execution, and return. The time of offloading can be expressed as follows:

(3) $$t_{u,v}^{up}=\frac{\varepsilon D_{u}}{r_{u,v}}$$

where ε is the proportion of tasks offloaded to edge nodes.

$f_{u,v}$ represents the computing resources allocated to UAV u by edge node v. The execution time can be expressed as follows:

(4) $$t_{u,v}^{exe}=\frac{\varepsilon \alpha_{u}D_{u}}{f_{u,v}}$$

The calculation results are transmitted back to the UAV through the downlink channel of the OFDMA system. It is assumed that the return result of the task is very small compared with the input data, and the return time can be ignored [28]. Therefore, completion time $t_{u,v}^{mec}$ can be represented as follows:

(5) $$t_{u,v}^{mec}=t_{u,v}^{up}+t_{u,v}^{exe}$$

The completion time of the task, $t_{u}^{complete}$, depends on the latest completion time in the edge node, which can be expressed as follows:

(6) $$t_{u}^{complete}=\max\{t_{u}^{local},t_{u,1}^{mec},t_{u,2}^{mec},\cdot \cdot \cdot ,t_{u,m}^{mec}\}$$

When the task is offloaded to the edge node for calculation, the energy consumption only considers the consumption during transmission, so the energy consumption [28] can be expressed as follows:

(7) $$E_{u,v}^{up}=P_{u}^{T}t_{u,v}^{up}$$

where $P_{u}^{T}$ indicates the transmission power of UAV u.

The energy consumption [29] of the edge server when calculating tasks can be expressed as follows:

(8) $$E_{v}^{edge}=\kappa \sum_{u=1}^{n}f_{u,v}^{2}t_{u,v}^{exe}$$

where κ represents the coefficient of the CPU energy structure, which is used to calculate the energy consumption of task computing.

### 2.6. Utility Function

The utility function of edge nodes can be expressed as follows:

(9) $$U_{v}^{edge}=p_{v}\sum_{u=1}^{n}f_{u,v}-eE_{v}^{edge}$$

where e represents the price of unit electricity, and $p_{v}$ denotes the resource price of edge node v.

If the price of the edge node is too low, even if all computing resources are sold, it does not obtain satisfactory income and disrupts the market, resulting in other edge nodes following suit and reducing prices, such that none of the edge nodes obtains high income. If the price is too high, most UAVs tend to buy other low-cost computing resources instead of the edge node’s computing resources, which leads to low benefits for this edge node. Therefore, edge nodes should set an appropriate price to obtain satisfactory income. In order to obtain the optimal utility of edge node v, the optimal pricing strategy is $p_{v}^{*}=\arg\max U_{v}^{edge}(p_{v},p_{-v}^{*},F^{*})$, where $p_{-v}^{*}$ represents the optimal strategy of edge nodes other than edge node v, and $F^{*}$ is the optimal purchasing strategy of computing power for each UAV. The goal of the edge node is to maximize its utility function, that is, $\max U_{v}^{edge},v\in V$.

The benefit of UAVs brought by time can be measured by time $t^{save}$. When a task is calculated locally, the task completion time is $t^{all\_local}$. When UAV u offloads a task to edge server v for calculation, the task completion time can be expressed as $t_{u,v}^{mec}$. Local execution time minus the average completion time of each offloaded subtask represents the average time that can be saved after offloading, which is as follows:

(10) $$t_{u}^{save}=t_{u}^{all\_local}-\frac{t_{u}^{local}+\sum_{v=1}^{m}t_{u,v}^{mec}}{m+1}$$

Obviously, in order to increase $t^{save}$, UAVs have to continue to purchase computing resources, which causes a waste of resources. To avoid this, we take $U_{time}=\ln\left(1+t^{save}\right)$, representing the benefit of UAVs brought by time. $U_{time}$ increases with the increase in $t^{save}$. With the continuous purchase of resources by UAVs, $U_{time}$ conforms to the law of diminishing marginal utility; even if more time is saved, $U_{time}$ does not increase much.

The resource consumption of each UAV includes energy consumption during data transmission and MEC calculation resource consumption. Therefore, the total resource consumption can be expressed as follows:

(11) $$U_{u}^{pay}=\omega \sum_{v=1}^{m}E_{u,v}^{up}+\sum_{v=1}^{m}p_{v}f_{u,v}$$

where ω represents the cost coefficient of energy consumption during transmission.

Therefore, the utility function of any UAV can be formulated as follows:

(12) $$\begin{array}{l} U_{u}^{uav}=aU_{{}_{u}}^{time}-bU_{{}_{u}}^{pay}+cU_{{}_{}}^{success}\\ \;\;\;\;\;\;\;\;\;\;=a\ln(1+t_{u}^{all\_local}-\frac{t_{u}^{local}+\sum_{v=1}^{m}t_{u,v}^{mec}}{m+1})\\ \;\;\;\;\;\;\;\;\;\;\;\;\;\;-b(\omega \sum_{v=1}^{m}E_{u,v}^{up}+\sum_{v=1}^{m}p_{v}f_{u,v})+cU^{success}\\ \;s.t.:0\leq a\leq 1,0\leq b\leq 1,0\leq c\leq 1,a+b+c=1\\ \;\;c=\{\begin{array}{c} c\;,if\;\max\{\frac{\alpha D}{f_{total}},t_{1}^{mec},\ldots ,t_{m}^{mec},t_{1}^{cloud},\ldots ,t_{m}^{cloud}\}\leq t^{\max}\\ -c\;,if\;\max\{\frac{\alpha D}{f_{total}},t_{1}^{mec},\ldots ,t_{m}^{mec},t_{1}^{cloud},\ldots ,t_{m}^{cloud}\}>t^{\max}\; \end{array}\\ \;\;\;\;\;\;\;\;\;\;\;\;\;\;\;\;\;\;\;\;\;\;\;\;\;\;\;\;\;D>0\\ \;\;\;\;\;\;\;\;\;\;\;\;\;\;\;\;\;\;\;\;\;\;\;\;\;\;\;\;\;\alpha >0\\ \;\;\;\;\;\;\;\;\;\;\;\;\;\;\;\;\;\;\;\;\;\;\;\;\;\;\;\;\;y>0\\ \;\;\;\;\;\;\;\;\;\;\;\;\;\;\;\;\;\;\;\;\;\;\;\;\;\;\;\;\;\;u\in U,v\in V \end{array}$$

where a is the expenditure-sensitive factor; b is the time-sensitive factor; c is the mission-success-sensitive factor; $U^{time}$ represents the utility brought by the saved time when the UAV offloads a task to the MEC servers. $U_{success}$ represents the reward for task completion, which is a constant greater than 0. The UAV formulates its own optimal demand strategy according to the price strategy sent by the edge node. Due to the limited computing power of each edge node, the demand strategies among UAVs affect each other. The strategy set of all UAVs can be expressed as $F=(f_{1},f_{2},\ldots ,f_{n})$; the resource purchase strategy of UAV u is $f_{u}=(f_{u,1},f_{u,2},\ldots ,f_{u,m}),u\in U_{}$, while the optimal strategy can be expressed as $f_{u}^{*}=\arg\max U_{u}^{uav}(P^{*},f_{u},f_{-u}^{*})$, where $f_{-u}^{*}$ represents the optimal strategy of UAVs other than UAV u. The objective of each UAV is to maximize the utility function, that is, $\max U_{u}^{uav},u\in U$.

## 3. Problem Formulation

In this section, for task offloading in a UAV network, we model the maximizing utility problem in $\max U_{u}^{uav},u\in U$ and $\max U_{v}^{edge},v\in V$ as an MDP by defining state space S, action space A, and reward function R. The agents are divided into the following two layers: leader and follower, in which the leader is the edge node, and the follower is the UAV. It is assumed that the two-tier agents in the game process are asymmetric; that is, the follower agent observes the behavior of the leader agent so as to solve the two-tier optimization problem of the Markov game.

Therefore, the game problem in this paper can be described as follows:

(13) $$\begin{array}{l} \underset{\pi_{1}}{\max}\;E_{r_{1}^{1},r_{1}^{2}\ldots \sim a_{l},a_{f}}\sum_{t=1}^{T}\gamma^{t}r_{1}^{t}\\ s.t.a_{l}\in A_{l}\\ \;\;\;\;\;\;\underset{\pi_{2}}{\max}\;E_{r_{2}^{1},r_{2}^{2}\ldots \sim a_{l},a_{f}}\sum_{t=1}^{T}\gamma^{t}r_{2}^{t}\\ \;\;\;\;\;\;s.t.a_{f}\in A_{f} \end{array}$$

where $a_{l},a_{f}$ indicate the actions of leaders and followers, respectively. $r^{1},r^{2}$ represent the rewards of leaders and followers, respectively. γ represents the reward discount rate of the agent, which is used to measure the importance of future rewards and current rewards, and its value range is [0,1]. The closer γ is to 1, the more important the future reward is. The whole game can be represented by octets $<s_{l},s_{f},a_{l},a_{f},{s^{\prime }}_{l},{s^{\prime }}_{f},r_{l},r_{f}>$. In an environment with M+N agents, $\pi =(\pi_{1},\pi_{2},\ldots ,\pi_{m+n})$ represents the strategies of all agents; $\theta =(\theta_{1},\theta_{2},\ldots ,\theta_{m+n})$ represents the strategy parameters of agents; $\mu =\left(\mu_{1},\mu_{2},\ldots ,\mu_{m+n}\right)$ represents the deterministic strategies of agents.

### 3.1. State Space

The state space of each leader agent and follower agent at time t is defined respectively as follows:

(14) $$s_{t}^{leader}=\left\{i\left(t\right),f_{free}\left(t\right),u\left(t\right)\right\}$$

(15) $$s_{t}^{follower}=\left\{i\left(t\right),P_{mec}\left(t\right),T\left(t\right),R\left(t\right)\right\}$$

where $f_{free}(t)$ represents the available computing resources of the computing node at time t; u(t) represents the resource utilization of each UAV at time t; i(t) represents edge node information; $P_{mec}(t)$ is the decision set of leaders at time t, and T(t) is the concurrent task information set at time t, including task size, latest completion time, time-sensitive factor, price-sensitive factor, etc. R(t) is the set of data transmission rate at time t.

### 3.2. Action Space

The action spaces of the leader and follower are as follows:

(16) $$a_{t}^{leader}=\left\{p(t)\right\}$$

(17) $$a_{t}^{follower}=\left\{f(t)\right\}$$

where p(t) represents the price of unit computing resources of the edge node at time t; f(t) represents the collection of computing resources purchased by the UAV from each edge node.

### 3.3. Reward Function

The reward functions of the leader and follower are as follows:

(18) $$r\left(s_{t}^{leader},a_{t}^{leader}\right)=U_{mec}$$

(19) $$r\left(s_{t}^{follower},a_{t}^{follower}\right)=U_{uav}$$

The goal of the system is to obtain the offloading strategy to maximize the cumulative leader utility under the condition of maximizing the cumulative follower utility. The cumulative reward of each agent, that is, the objective function is as follows:

(20) $$J(\theta )=\max E\left[\sum_{t=0}^{T}\gamma^{t}r(s_{t},a_{t})\right]$$

## 4. Task Offloading Based on RL

In this section, we first review the basic theoretical knowledge of reinforcement learning (RL). Then we introduce the MADDPG algorithm model. Finally, the training process of the agent based on the MADDPG algorithm for task offloading is introduced, and the corresponding pseudo-code is shown.

### 4.1. Reinforcement Learning

RL is the third learning method in machine learning, besides supervised learning and unsupervised learning. The characteristic of reinforcement learning is that, without given training data in advance, it uses environmental feedback as input to learn by constantly trying and correcting its own strategies in the environment, and it makes the agent learn the best or approximate the optimal solution in the environment by maximizing the cumulative reward expectation.

**Figure 3:**
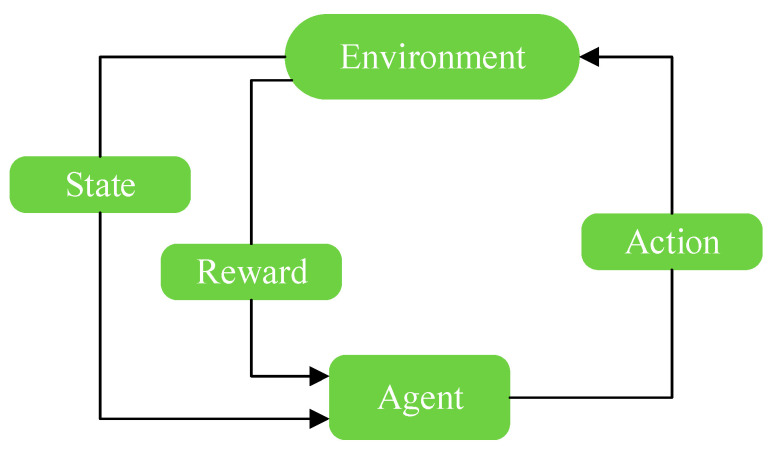
Interaction process between agent and environment.

As shown in Figure 3, the agent executes an action $a_{t}$ in the environment according to the current state $s_{t}$ at time t; after-action $a_{t}$ occurs, the environment changes, the current state $s_{t}$ shifts to the next state, $s_{t+1}$, and the agent obtains reward $r_{t}$ from the environment. According to the new environmental state, $s_{t+1}$, the agent executes action $a_{t+1}$ and obtains reward $r_{t+1}$. This loop runs until the end state of the environment, and the agent completes a complete interaction process in the environment. The purpose of the agent is to find a strategy that can maximize the cumulative reward function.

### 4.2. MADDPG Algorithm Model

The MADDPG algorithm is a natural extension of the DDPG algorithm under the multi-agent system. It belongs to centralized training and has an algorithm framework for decentralized execution. The MADDPG algorithm has made a series of improvements based on the Actor-Critic algorithm and the DDPG algorithm; it adopts the principle of centralized learning and distributed application, which makes it suitable for the complex multi-agent environment that the traditional reinforcement learning algorithm cannot deal with. Traditional reinforcement learning algorithms must use the same information data in learning and application, while the MADDPG algorithm allows some additional information (i.e., global information) to be used in learning, but only local information is used in application decisions. Compared with the traditional actor-critical algorithm, there are M+N agents in the MADDPG algorithm environment. The strategy of agent i is represented by $\pi_{i}$, and its strategy parameter is $\theta_{i}$; then, the strategy set of M+N agents is $\pi =\pi_{1},\pi_{2},\ldots ,\pi_{m+n}$, and the set of strategy parameters and actions are $\theta =\theta_{1},\theta_{2},\ldots ,\theta_{m+n}$ and $a=a_{1},a_{2},\ldots ,a_{m+n}$. The cumulative expected return of agent i is as follows:

(21) $$J\left(\theta \right)=E_{s\sim p^{\pi },a_{i}\sim \pi_{\theta_{i}}}\left[\sum_{\;t=0}^{\infty }\gamma^{t}r_{i,t+1}\right]$$

where $r_{i,t}$ represents the reward obtained by agent i at time t. In the multi-agent environment, we mainly consider the rewards of different agents at the same time, so we replaced $r_{i,t}$ with $r_{i}$. $p^{\pi }$ is the state distribution under strategy π. $\pi_{\theta_{i}}\left(a|s\right)$ is a random strategy function used to map the probability distribution from state to action. t represents the time in the environment.

$O_{i}$ represents the observation value of agent i, and $x=o_{1},o_{2},\ldots ,o_{m+n}$ represents the observation vector. $Q_{i}^{\mu }\left(x,a_{1},a_{2},\ldots ,a_{m+n}\right)$ is a centralized state action function, which includes not only the observed states and actions, but also $\left(a_{1},\;a_{2},\ldots ,a_{m+n}\right)$, which represent the actions of other agents. Then, in the random strategy, the strategy gradient formula can be obtained as follows:

(22) $$\nabla_{\theta_{i}}J\left(\theta \right)=E_{s\sim P_{\pi },a_{i}\sim \pi_{\theta_{i}}}\left[\nabla_{\theta_{i}}ln\pi_{i}\left(a_{i}|o_{i}\right)Q_{i}^{\mu }\left(x,a\right)\right]$$

Therefore, the critical network of each agent knows not only the changes of its own agent but also the action strategies of all other agents.

In order to improve the problem of low convergence efficiency when selecting actions according to probability, MADDPG algorithm was extended to deterministic strategy. Let the continuous deterministic strategy of M+N agents be $\mu_{\theta_{i}}$, and its return expectation gradient is as follows:

(23) $$\nabla_{\theta_{i}}J\left(\mu_{i}\right)=E_{x,a∼D}\left[\nabla_{\theta_{i}}\mu_{i}\left(a_{i}|o_{i}\right)\nabla_{a_{i}}Q_{i}^{{}^{\mu }}\left(x,a\right){|}_{a_{i}=\mu_{i}\left(o_{i}\right)}\right]$$

where D is the experience pool, which stores the experience of all agents. Each sample datum is composed of $\left(x,x\prime ,a_{1},a_{2},\ldots ,a_{m+n},r_{1},r_{2},\ldots ,r_{m+n}\right)$. $Q_{i}^{\mu }$ is the action value function. $Q_{i}^{\mu }$ establishes a value function for each agent, which greatly solves the shortcomings of the traditional reinforcement learning algorithm in the field of multi-agent environments. The updated formula of $Q_{i}^{\mu }$ is as follows:

(24) $$L\left(\theta_{i}\right)=E_{x,a,r,x^{\prime }}\left[{\left(y-Q_{i}^{\mu }\left(x,a_{1},\cdots ,a_{m+n}\right)\right)}^{2\;}\right]$$

where y is obtained from the following formula:

(25) $$y=r_{i}+\gamma {\bar{Q}}_{i\;}^{\mu \prime }\left(x\prime ,a_{1}^{\prime },\cdots ,a_{m+n}^{\prime }\right){|}_{a_{j}^{\prime }=\mu_{j}^{\prime }\left(o_{j}\right)}$$

where ${\bar{Q}}_{i}^{\mu \prime }$ represents the target network, $a_{j}^{\prime }=\mu_{j}^{\prime }\left(o_{j}\right)$ represents the predicted action by the target actor network and $\mu \prime =\left[\mu_{1}^{\prime },\mu_{2}^{\prime },\cdots ,\mu_{m+n}^{\prime }\right]$ is the parameter of the target strategy with lag update.

${\overset{⌢}{\mu }}_{\phi_{i}^{j}}$ represents the approximation function of agent i to deterministic strategy $\mu_{j}$ of agent j. The approximation cost is a logarithmic cost function, and with the entropy of the strategy, the cost function can be written as follows:

(26) $$L\left(\phi_{i}^{j}\right)=-E_{o_{j},a_{j}}\left[ln{\overset{⌢}{\mu }}_{\phi_{i}^{j}}\left(a_{j}|o_{j\;}\right)+\lambda H\left({\overset{⌢}{\mu }}_{\phi_{i}^{j}}\right)\right]$$

As long as the above cost function is minimized, the approximation of other agent strategies can be obtained. Therefore, y can be changed to the following:

(27) $$y=r_{i}+\gamma {\bar{Q}}_{i}^{\mu \prime }\left(x\prime ,{\overset{⌢}{\mu }}_{\phi_{i}^{j}}^{1}\left(o_{1}\right),\ldots ,\;{\overset{⌢}{\mu }}_{\phi_{i}^{j}}^{m+n}\left(o_{m+n}\right)\right)$$

Before updating $\phi_{i}^{\mu }$, a sampling batch of experience reply is used to update the approximation function of agent i to deterministic strategy $\mu_{j}$ of agent j. In this way, the purpose of other agent strategies can be obtained by fitting the approximation without communicating with each other. The core idea of the algorithm is that each agent has its own strategy network. The evaluation network uses the experience of each agent and combines state actions as input. For the strategy network, only the observation value and state information of the agent are used in the training. For the evaluation network, it is only used in the network training. The information used includes the states and actions of all agents, and the corresponding Q value is output.

In the process of updating the network, a batch of data at the same time are randomly extracted from the experience pool of each agent and spliced to obtain new experience 〈S,A,S′,R〉, where S and S′ are the state combinations of all agents at the same time; A is the set of actions made by all agents at the same time; R selects the return value of agent i. Finally, input S′ into the target strategy network of agent i to obtain action A′; then, we input A′ and S′ together into the target evaluation network of agent i to obtain the value of target Q estimated for the next time, and calculate the value of target Q at the current time according to the following formula:

(28) $$y\prime =r_{i}+\gamma Q^{\prime }\;\left(s_{i+1},\mu \prime \left(s_{i+1}|Q^{\mu \prime }\right)|\theta^{Q^{\prime }}\right)$$

The actual value of Q is obtained by using the evaluation network; then, the TD deviation [30] is used to update the evaluation network, and the strategy gradient of Q is used to update the strategy network. All agents update their networks in the same way, but the input of each agent is different, and the update process is the same under other aspects.

### 4.3. Task Offloading Algorithm Based on MADDPG

To balance the delay and consumption of the UAV and achieve the goal of maximizing the overall system utility, we propose an experience-driven offloading strategy based on multi-agent reinforcement learning. The algorithm can make effective offloading decisions without solving complex mathematical models, so as to make efficient use of computing resources. The algorithm is centrally trained on the task controller and then deployed to UAVs and edge nodes for distributed execution.

In this UAV network, there are M+N distributed deployment agents. Each agent independently observes its environment in parallel and interacts with the environment to obtain different states, selecting corresponding actions based on the state. For a single agent, first, we input its state into its own strategy network, obtain an action output, and then act on the environment. Then, a new state and return value are obtained. Finally, the agent stores the state transfer data into the agent’s own experience pool. All agents constantly interact with the environment and constantly generate data and store them in their own experience pool.

The specific description of the MADDPG task offloading algorithm based on the Stackelberg model is shown in Algorithm 1.
**Algorithm 1. MADDPG task offloading algorithm based on Stackelberg model**$\mathrm{Input}:\;\mathrm{Set}\;\mathrm{of}\;\mathrm{UAV}\;U=\left\{u_{1},u_{2},\cdot \cdot \cdot ,u_{n}\right\}$$,\;\mathrm{initial}\;\mathrm{parameters}\;\mathrm{of}\;\mathrm{UAV}\;\langle x_{0},y_{0},t,f_{local}\rangle$$,\;\mathrm{and}\;\mathrm{edge}\;\mathrm{network}\;\mathrm{environment}\;\mathrm{parameters}\;\left(L_{site},R_{u,v},B,P_{u,v},N,h,\theta_{1},\theta_{2}\right)$. Steps of each episode and number of episodes;

Output: Total utility function value of each UAV and system at each episode;

1. Initialize the edge node environment and UAV environment according to the edge network environment parameters;
2. Initialize parameters in leader_network and follower_network; initialize the space of $D_{l}$$,\;D_{f};$
3.  for episode=1: n_episode do

4.    for step=1: steps do

5.      Calculate the uplink rate of UAV according to formula (1); # Obtain leader state and follower state;
$6.\;\;\;\;\;s_{l}$$=\mathrm{leader}\_\mathrm{env}.\mathrm{getState}();s_{f}=\mathrm{follower}\_\mathrm{env}.\mathrm{getState}();$7.      Update position of UAV; # Get leader action and follower action, and add noise to the action to ensure the exploration rate;
$8.\;\;\;\;\;a_{l}$$=\mathrm{leader}\_\mathrm{network}.\mathrm{action}(s_{l}$$);\;a_{f}$$=\mathrm{follower}\_\mathrm{network}.\mathrm{action}\;(s_{f});$$9.\;\;\;\;\;a_{l}$$+=\mathrm{noise};\;a_{f}+=\mathrm{noise};\;\#\;\mathrm{Obtain}\;\mathrm{reward}\;\mathrm{from}\;\mathrm{leaders}\;\mathrm{and}\;\mathrm{followers};$$10.\;\;\;\;r_{f}$$,\;s_{f}\prime$$=\mathrm{follower}\_\mathrm{env}.\mathrm{step}\;(a_{f}$$,\;a_{l});$$\;\;r_{l}$$,s_{l}\prime$$=\mathrm{leader}\_\mathrm{env}.\mathrm{step}\;(a_{l}$$,\;a_{l});\;$$\mathrm{where}\;s_{f}\prime ,s_{l}\prime \;\mathrm{represent}\;\mathrm{the}\;\mathrm{next}\;\mathrm{states}\;\mathrm{of}\;\mathrm{follower}\;\mathrm{and}\;\mathrm{leader},\;\mathrm{respectively};\;$      # Storage experience;
11. $D_{l}$$.\;\mathrm{Store}\;(s_{l}$$,a_{l}$$,r_{l}$$,s_{l}\prime );$
$\;\;\;\;D_{f}$$.\;\mathrm{Store}\;(s_{f}$$,a_{f}$$,r_{f}$$,s_{f}\prime );$$\mathrm{where}\;D_{l},D_{f}\;\mathrm{represents}\;\mathrm{the}\;\mathrm{experience}\;\mathrm{pool}\;\mathrm{of}\;\mathrm{leader}\;\mathrm{and}\;\mathrm{follower},\;\mathrm{respectively}.$12. $\;\;\;\;\;\mathrm{leader}\_\mathrm{network}.\mathrm{learn}\;(D_{l}$$);\;\mathrm{follower}\_\mathrm{network}.\mathrm{learn}\;(D_{f});$
13.     Update network parameters;
14. $\;\;\;\;\;s_{l}$$=s_{l}\prime$$;\;s_{f}$$=s_{f}\prime ;\;\#\mathrm{Assign}\;\mathrm{the}\;\mathrm{next}\;\mathrm{state}\;\mathrm{to}\;\mathrm{the}\;\mathrm{current}\;\mathrm{state};$
15.    end
16.  end

## 5. Performance Evaluation

### 5.1. Experiment Environment

In this section, the offloading algorithm based on reinforcement learning proposed in this paper is simulated, and the performance of the algorithm is analyzed. The simulation environment was Python 3 8.5. The length of the system site was 100 m; the UAVs were distributed near the edge calculation nodes; the altitude of the UAVs was h = 20 m; the UAVs were randomly distributed. There were five evenly distributed edge nodes in the system environment. The price sensitivity coefficient, delay sensitivity coefficient, mission-success or -failure sensitivity coefficient, initial position, and speed of each UAV were generated randomly. The channel parameters were the following: the bandwidth was 100 MHz, and the Gaussian white noise power was 2.5 × 10 ^−13^ w. For the UAV, its computing power was 1.5 GHz. The volume of each task was a random value in the range of 100~150 MB. The computing power of the edge nodes was 10 GHz.

The parameters, definitions, and values of the simulation experiment are shown in the Table 1.

### 5.2. Simulation Results and Analysis

**Figure 4:**
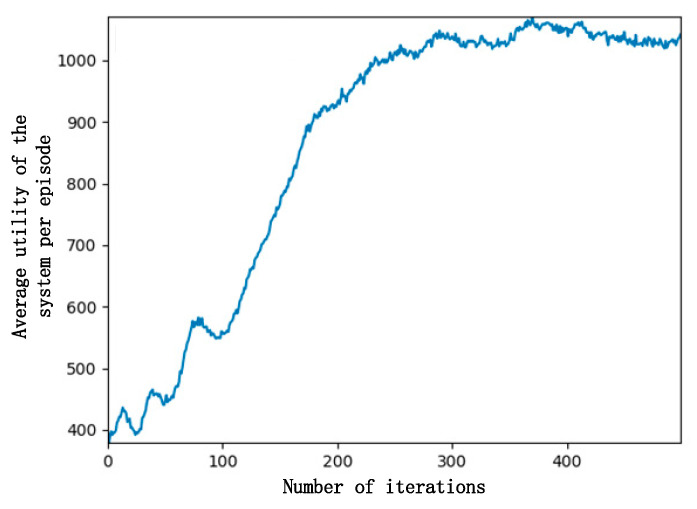
Curve of system utility and number of iterations.

Figure 4 demonstrates how the number of iterations affects the total utility of the system. The total utility of the system is defined as the weighted sum of the standardized rewards of leaders and followers. It can be seen from the figure that in about 80 iterations, the total utility of the system reached a maximum, but the leader utility and follower utility were not balanced, and neither accepted such a result, so both sides learned from previous experience and then adjusted the strategy.

At the 80th–100th iterations, the strategy began to be adjusted, and the total utility of the system decreased. This is because the leader started to raise the selling price, and the follower fine-tuned the purchase decision; then, the follower utility decreased, and the leader utility increased too much. After the first 300 iterations, the average utility of the system continued to increase and then gradually stabilized at about 1000 after 300 iterations, almost reaching convergence and better completing the task of maximizing the utility of the system.

**Figure 5:**
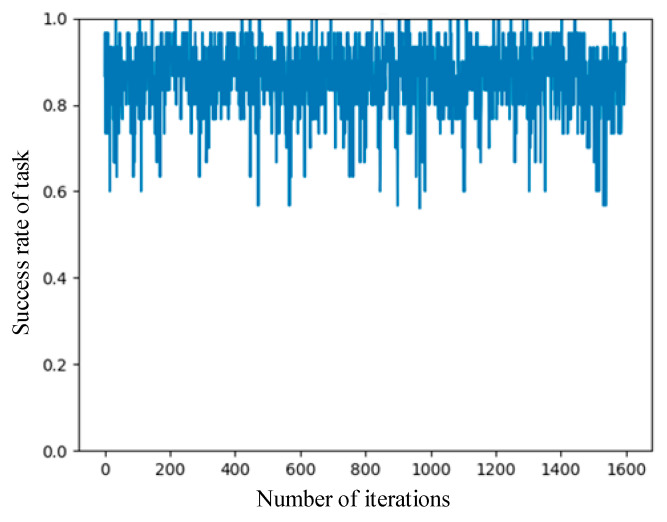
Curve of success rate of task.

Figure 5 shows the curve of the success rate of the UAV mission with respect to the number of episode iterations when the numbers of leaders and followers are 5 and 15, respectively. It can be seen from the figure that the UAV mission success rate was unstable; it fluctuated back and forth between 80% and 90%, with the lowest success rate being about 60% and the highest success rate being 100%. The main reason for the fluctuation in the mission success rate is that the mission-success-sensitive factor c of the UAV was generated randomly.

Some UAVs were not sensitive to whether the task could be completed on time, so the value of c was low. Even if the task completion time exceeded the delay, UAVs could still obtain high utility. Moreover, the volume of the task was 100~150 MB, and the task completion delay was 1–5 s. Assuming that the data size of the task is 150 MB, and the task completion delay is less than 1 s, if UAVs purchase too many computational resources to compute tasks, the utility of the UAVs is reduced. The experiments show that the offloading algorithm proposed in this paper can maintain a high task success rate.

In order to verify the effectiveness of the MADDPG in the UAV network, the proposed algorithm was compared with the following typical strategies:

(1) NSGA (non-dominated sorting genetic algorithms) multi-objective genetic algorithm: The decisions of purchase power and fix price are made simultaneously by the algorithm, and the Pareto optimal solution of purchase power decision and fix price decision is obtained;
(2) Random algorithm: The purchasing decision of computing power is generated randomly, and the offloading proportion is generated randomly;
(3) QoS priority algorithm: It distributes all tasks equally to all edge nodes and minimizes task delay as much as possible.

**Figure 6:**
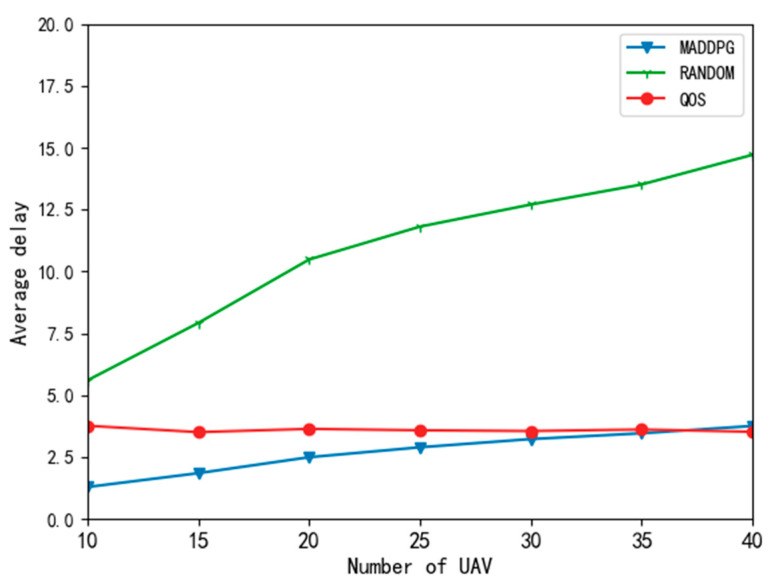
Average delay curve of different algorithms.

Figure 6 compares the impact of the different algorithms on task delay when changing the number of UAVs. It can be seen from the simulation diagram that the MADDPG algorithm could significantly reduce the task delay. The main reason is that the random algorithm randomly offloads the tasks of the UAV to the edge node, does not consider the resource state of the edge node and UAV in the system, and cannot make full use of the computing resources of local and edge nodes. This also leads to other UAVs being unable to obtain the offloading decision with the best utility, so the average delay of the task is the highest. With the increase in the number of UAVs, the average resource decreased, resulting in an increase in the average delay. When the number of UAVs grows larger than 35, the average delay of the MADDPG exceeds that of the QoS priority algorithm. The reason is that the QoS priority algorithm offloads all tasks equal to the edge nodes and does not consider utility. Moreover, the edge nodes have enough computing resources to compute these tasks, so the average delay of the QoS priority algorithm could be kept low. Although its average delay was lower than that of the MADDPG algorithm, the QoS priority algorithm could not make rational use of the computing resources of local and edge nodes. Still, the MADDPG algorithm could obtain a low average delay.

**Figure 7:**
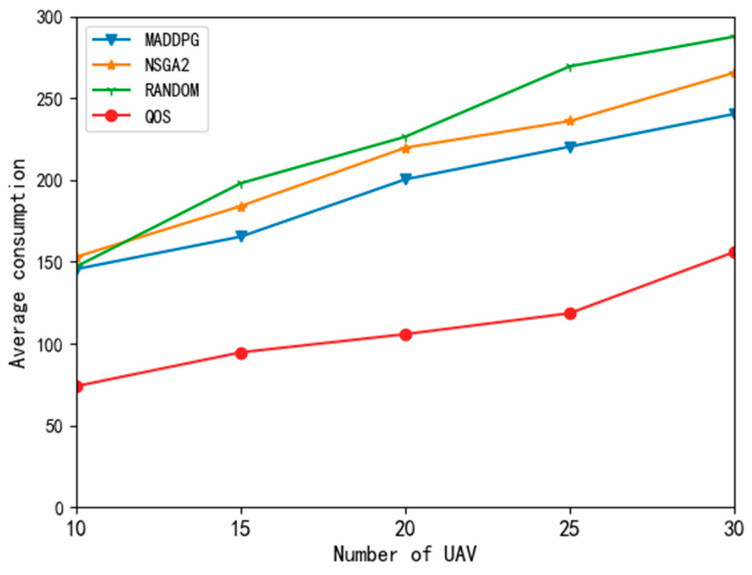
Average energy consumption curve of different algorithms.

Figure 7 shows the energy consumption curves of different algorithms. Because the QoS algorithm offloads all tasks to the edge nodes, the UAV does not need to calculate tasks and only considers transmission energy consumption, so the energy consumption is quite low. The volume of edge node resources remains unchanged; with the increase in the number of UAVs, edge nodes increase the price of the resource, and UAVs begin to change their strategies and execute more tasks locally, so the average energy consumption also begins to increase.

When the number of UAVs was 10, the average energy consumption of the random algorithm, NSGA2 algorithm, and MADDPG algorithm was similar. When the number of UAVs exceeded 15, the average energy consumption of the MADDPG algorithm was significantly lower than that of the other two algorithms. It can be seen that the MADDPG algorithm could better reduce the average energy consumption of UAVs compared with the random algorithm and the NSGA2 algorithm.

**Figure 8:**
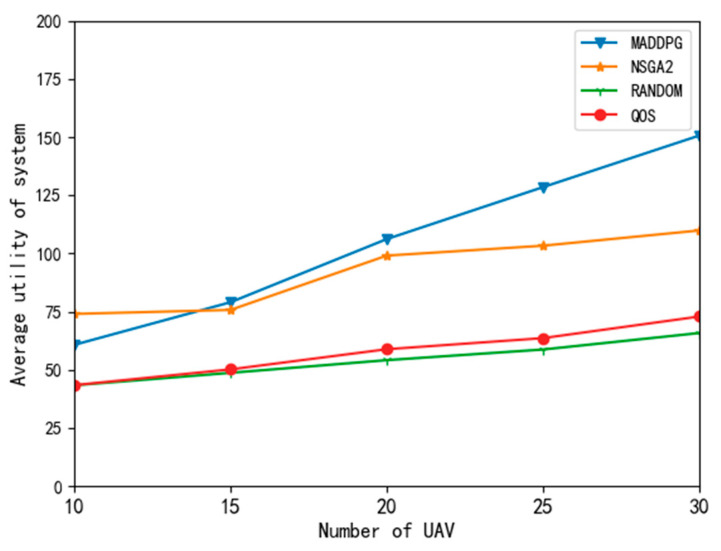
Average utility curve of system with different algorithms.

Figure 8 shows the comparison results of average utility with different algorithms. The results show that the MADDPG algorithm proposed in this paper could maximize the average utility of the system. The total system utility in this paper is the weighted sum of the average utility of the UAVs and the average utility of the edge nodes. It can be seen from the figure that the QoS and random algorithms performed poorly, mainly because they do not comprehensively consider delay, expenditure, and task timeout penalties.

The MADDPG algorithm obtained a high total system utility. The increase in the number of UAVs allowed it to make better use of the resources in the system, so the system utility was improved. When the number of UAVs is 10, the utility value of the MADDPG algorithm is lower than that of the NSGA2 algorithm, which evolved over 2000 generations. When the number of UAVs increased to 15 or above, the MADDPG algorithm performed better than the NSGA2 algorithm.

**Figure 9:**
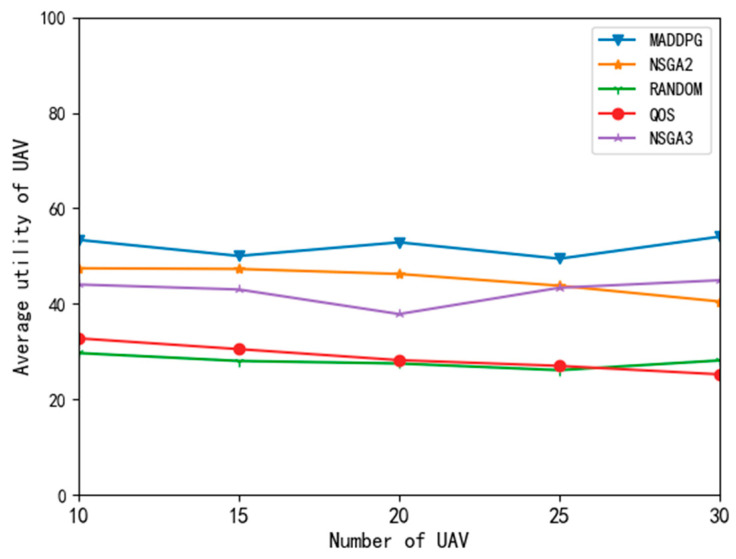
Average utility curve of UAV with different algorithms.

Figure 9 shows the comparison results of UAV average utility with different algorithms. On the average utility curve of UAVs, the QoS and random algorithms performed mediocrely. This is because the QoS algorithm only pays attention to the task processing delay, blindly reduces the task delay, and ignores the impact of expenditure on utility value. These two algorithms are extreme and can not achieve the compromise of delay, energy consumption, and task success or failure reward. The MADDPG algorithm performed well. The average utility of the UAVs with the MADDPG did not decrease with the increase in the UAV number, and the utility was higher than that obtained with the NSGA2 and NSGA3 algorithms.

Because the MADDPG algorithm in this paper is a two-tier structure with sequential actions, the UAV (follower) makes decisions according to the decisions of the edge node (leader) and estimates the strategies of other agents at the same level to make decisions to maximize its own utility. Therefore, this algorithm can achieve a good compromise between delay, energy consumption, and task success rate and obtain a large task utility value.

## 6. Conclusions

In the UAV network based on MEC, we optimize task offloading by analyzing the delay and energy consumption of UAVs, so as to maximize the total utility of the UAV patrol system. According to the MADDPG algorithm and the Stackelberg game model, this paper proposes the Stackelberg MADDPG algorithm to solve the problem of task offloading. The MADDPG algorithm proposed in this paper relies on historical task information for learning and has low dependence on the optimization model. Not only can it effectively solve the nonconvex optimization problem of the UAV utility function, but it can also meet the requirements of distributed computing. The proposed algorithm was simulated and evaluated. In the simulation experiment, we verified the performance of the algorithm by simulating the change in system utility with the number of UAVs and the number of iterations. The experimental results show that compared with the comparison algorithms, the algorithm proposed in this paper has a high task success rate, can effectively reduce the task delay, balance the delay and consumption of UAVs, and improve the utility of UAVs and the system.

## Figures and Tables

**Table 1:** Experimental parameters of MADDPG task offloading algorithm.

Parameter	Definition	Setting
$L_{width}$	Length of scene	100 m
$P_{v2v}$	Power of communication	20 W
B	Bandwidth	100 MHz
$f_{local}$	Computing power of UAV	1.5 GHz
$f_{s}$	Computing power edge node	10 GHz
N	Power of Gaussian white noise	2.5 × 10 ^−13^ w
h	Fading factor of transmission channel	4
D	Volume of task data	100~150 MB
$t_{\max}$	Latest completion time of task	5~20 s
k	Coefficient of CPU energy structure	$10^{-28}$
a	Expenditure-sensitive factor	0~0.5
b	Time-sensitive factor	0~0.5
